# Supplementary material for: Contribution and Interaction of Shiga Toxin Genes to Escherichia coli O157:H7 Virulence
Source: Toxins (Basel). 2019 Oct 18;11(10):607. doi: 10.3390/toxins11100607 (PMC6832461; doi:10.3390/toxins11100607)
Supplement: Supplementary file 1 [file toxins-11-00607-s001.pdf]

Supplementary Materials: Contribution and Interaction of Shiga Toxin Genes to *Escherichia coli* O157:H7 Virulence

Gillian A.M. Tarr, Taryn Stokowski, Smriti Shringi, Phillip I. Tarr, Stephen B. Freedman, Hanna N. Oltean, Peter M. Rabinowitz, and Linda Chui

1. Supplemental Methods

1.1. Covariate Adjustment

We adjusted our final risk difference (RD) and relative risk (RR) estimates for age, blood in the stool, vomiting, and fever. We considered age to confound the association between *stx* genotype and hemolytic uremic syndrome (HUS) (Figure 1). There is a relationship between age and *stx* genotype, with younger children more likely to be reported with *stx2a*-only and *stx2a2c* genotypes than *stx1a2a* and other genotypes. The *stx2a*-only and *stx2a2c* genotypes may be more capable of infecting and causing disease in young children, or conversely, worse at infecting and causing disease in older children and adults, thereby skewing the distribution of younger cases. There may also be differences in exposures. Particular *E. coli* O157:H7 strains may be disproportionally associated with some transmission vehicles than others [1], and transmission vehicles are possibly non-randomly distributed across ages. For example, cases infected with cattle-biased lineages of *E. coli* O157:H7 tend to be older, likely reflecting the greater contact between working-age individuals and cattle. Children <10 years, and especially those <5 years, are also at a substantially increased risk of HUS, thus causing confounding.

We adjusted for symptoms that may have been likely to alter how a patient was treated. Particular treatments may potentiate (e.g., antibiotics) or protect against (e.g., early intravenous fluids) the development of HUS. Some symptoms could make these treatments more or less likely. For example, bloody diarrhea may signal to a clinician a likely bacterial infection and they may treat with antibiotics. There is some evidence, including the current study, that symptoms like bloody diarrhea are more common with *stx1a2a* genotype strains. Therefore, there may be *E. coli* O157:H7 cases who receive antibiotics because of their bloody diarrhea, triggering progression to HUS, and this causal sequence may be more common in cases with the *stx1a2a* genotype (Figure 1). Lacking full data on interventions used, we adjusted for symptoms to block this alternate causal pathway.

A potential limitation of this approach is that our adjustment for symptoms may only be partially blocking the indirect causal pathway between genotype and HUS. We believe this likely had minimal impact on our estimates. Although bloody diarrhea cannot be explained entirely by *stx* genotype among non-O157 Shiga toxin-producing *E. coli* (STEC) [2], in our cohort bloody diarrhea was more commonly experienced by cases with the *stx1a2a* genotype. Conversely, vomiting was more commonly experienced by cases with the *stx2a* genotype. Hematochezia is often associated with bacterial agents and vomiting with viral agents [3], and therefore these symptoms may impact the likelihood of antibiotic treatment. Thus, the indirect path may be accounting for a larger portion of the *stx1a2a* genotype's than the *stx2a* genotype's HUS risk; fully controlling for it would be expected to increase RD and RR estimates.

1.2. Interaction

Stabilized inverse probability weights were calculated using a multinomial model that regressed genotype on age and symptoms. The probabilities p_{01}, p_{10}, and p_{11} were then calculated as the sum of the weights of HUS cases of the *stx2a*-only, *stx1a*-only, and *stx1a2a* genotypes, respectively, over the sum of the weights of all cases of the given genotype. The probabilities were then combined using the formula

$$p_{11} - p_{10} - p_{01} + p_{00} \quad (1)$$

with p_{00} set to 2/1,000,000 to obtain the amount of additive interaction. This entire process was bootstrapped, with 10,000 iterations, and bias-corrected and adjusted 95% confidence intervals were determined.

We did not assess multiplicative interaction. The risk of HUS in *E. coli* O157:H7 cases without any of the *stx* genes we tested for is near 0. With $p_{00} = 0$, all relative risks (RRs) are undefined and the relative excess risk due to interaction cannot be calculated.

1.3. Loss of stx Genes

Because of the potential for the loss of *stx*-carrying bacteriophages after isolation, we conducted a sensitivity analysis in which isolates with an atypical *stx* genotype for their pulsed field gel electrophoresis (PFGE) pattern and phylogenetic lineage [4] were reclassified to a different *stx* genotype. More than half of the isolates with a particular PFGE pattern needed to have the same *stx* genotype for us to reassign genotype. However, if the isolate had been typed to determine its phylogenetic lineage and it fell in a different lineage than the majority of isolates with the PFGE pattern, its *stx* genotype was not reclassified. For example, there were 11 isolates with the *Xba*I PFGE pattern EXHX01.0248. All were either typed to lineage Ib (n = 8) or did not undergo lineage typing (n = 3). Nine of these isolates had the *stx1a2a* genotype, one had the *stx1a*-only genotype, and one had the *stx2a*-only genotype. The latter two isolates were reclassified as having the *stx1a2a* genotype for the sensitivity analysis.

2. Supplemental Results

2.1. Missing Data

Of the 1,160 culture-confirmed cases reported to the department of health during the study period, 194 had no bacterial isolate in Washington State University's specimen bank or could not be revived. Over half of these (n = 125) were reported in the first three years of the study, 2005–2007. Missing predominantly the oldest isolates suggests that the most probable associated mechanisms are degradation and being misplaced. Neither of these is likely to be a function of genotype or HUS status. We believe they are largely random processes and satisfy criteria for missing completely at random.

We conducted supplemental analyses to determine if other factors appeared to affect isolate missingness. In univariate analysis, we found associations between missingness and presence of diarrhea, HUS status, hospitalization, and year. In multivariable analysis with these variables, only year [odds ratio (OR) 1.36; 95% confidence interval (CI) 1.27, 1.47] appeared to be associated with having an isolate available for testing. The point estimate for diarrhea was relatively high (OR 3.60), but the 95% CI (0.84, 13.9) suggested statistical uncertainty. Given the very small proportion of *E. coli* O157 cases without diarrhea, we do not believe this would have impacted our results.

We also examined patterns of missingness for HUS status. There were 28 cases with a genotyped isolate and 10 without a genotyped isolate for whom we could not determine HUS status. Case report forms for these cases indicated they were hospitalized, presenting the possibility that they could have experienced HUS. However, the indicated hospital either did not have a record for the patient during the time of the *E. coli* O157 episode (n = 36) or the record was incomplete and HUS status could not be determined (n = 2).

Among cases with a genotyped isolate, in univariate analysis, fever, having an underlying illness, year, and age were associated with missingness. After multivariable analysis, only year (OR 1.23; 95% CI 1.03, 1.48) and fever (OR 0.32; 95% CI 0.18, 0.83) were associated with having ascertained HUS status. A year in which illness occurred is likely a predictor of missingness, because older records may be more likely to have been lost, particularly for hospitals still using paper systems at the time. As with isolates, this was likely a random process. Cases with a fever were more likely to

have missing HUS status; 1% (8/545) of cases without a fever were missing HUS status vs. 4% (13/348) of those with a fever. These missing cases were evenly distributed across the genotypes. Given the similar distribution of fever across genotypes among known HUS status cases, these missing cases likely had little impact on the results.

2.2. Interaction

The small number of *stx1a*-only isolates compromised and yielded an estimate of additive interaction for *stx1a* and *stx2a* that lacked precision. The bootstrap generated a bimodal distribution, with the two peaks centered at −0.0396 and −0.270 (Figure S2). This suggests there may be heterogeneity but is more likely due to the small sample of *stx1a*-only isolates. There was one HUS case with a *stx1a*-only isolate, and in that case's stratum, defined by age and symptoms, there were no other *stx1a*-only isolates. Bootstrap samples that include this case are likely to overestimate the HUS risk associated with the *stx1a*-only genotype, making the interaction appear sub-additive to a very great degree. If the risk of HUS were 0 in cases with the *stx1a*-only genotype, we would expect the interaction to be the same as the RD between *stx1a2a* and *stx2a*; the −0.0396 peak is much closer to the RD, suggesting that the inclusion of the *stx1a*-only HUS case in a given bootstrap sample is driving the bimodal distribution. Only 152 of the 10,000 estimates of the interaction were ≥0.

2.3. Hospitalized Cases

We restricted this sensitivity analysis to cases who had been hospitalized. By definition, this removed only non-HUS cases, because non-hospitalized cases were assumed to not have HUS. Thus, HUS incidence increased for all genotypes. HUS incidence was 15.8% (28/177) for *stx1a2a*, 25.5% (24/94) for *stx2a*, and 18.8% (16/85) for *stx2a2c* hospitalized cases.

Given the much smaller sample size, RD and RR estimates were not adjusted for blood in stool and vomiting to avoid over-parameterizing. Both symptoms highly predicted antibiotic exposure, providing reassurance that adjusting for antibiotic use would block the indirect path. However, measured fever did not predict antibiotic use, though it did predict HUS. It may therefore be working through a different pathway and was left in the model.

3. Supplemental Figures

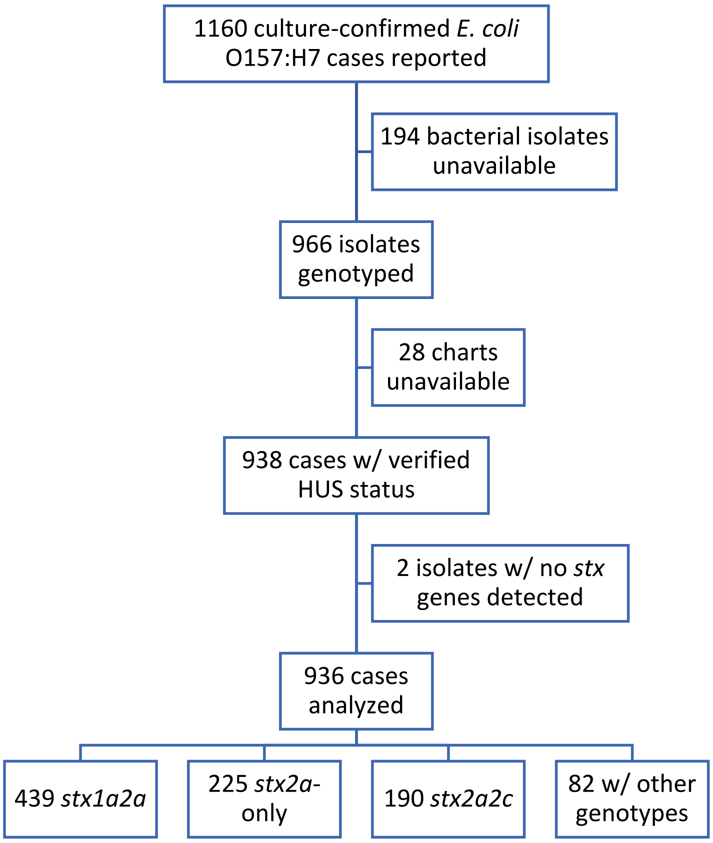

Figure S1. Flow diagram of *E. coli* O157:H7 cases and associated isolates included in analysis.

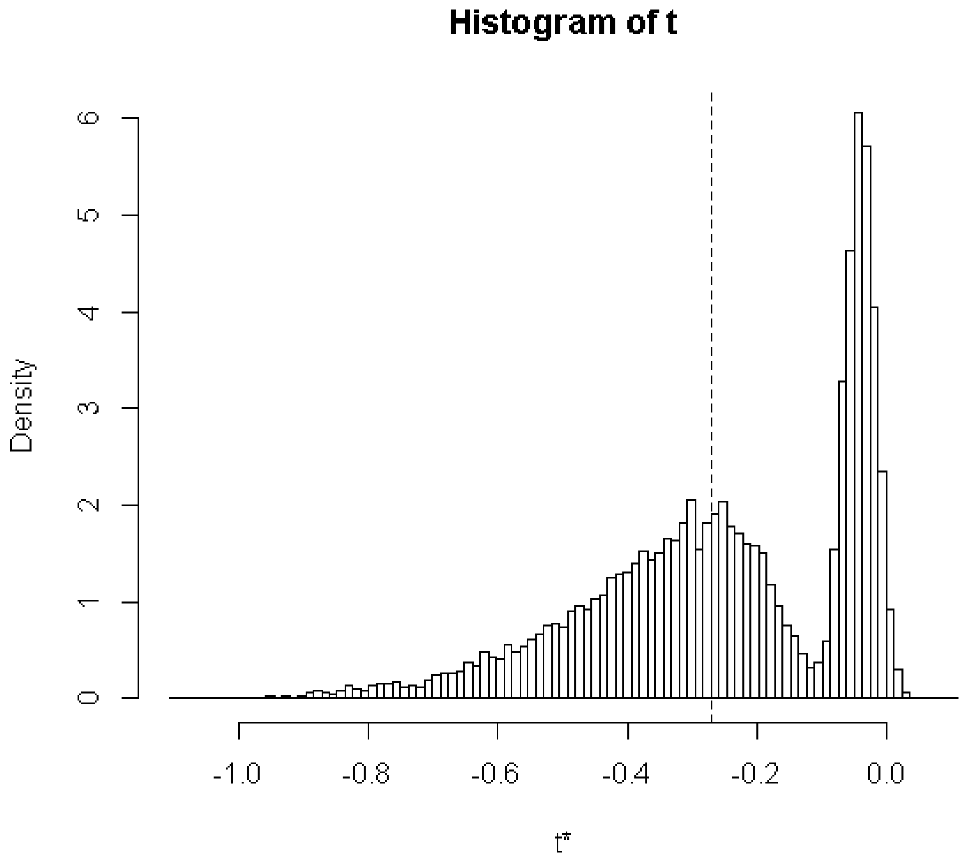

Figure S2. Histogram of 10,000 bootstrap samples for the interaction between *stx1a* and *stx2a*, showing a bimodal distribution of interaction estimates.

4. Supplemental Tables

Table S1. HUS status by age and *stx* genotype.

Age (years)	*stx1a2a*	*stx2a*	*stx2a2c*	Other
**<5**	15/96 (15.6%)	11/65 (16.9%)	8/58 (13.8%)	1/13 (7.7%)
**5–9**	6/65 (9.2%)	7/40 (17.5%)	4/25 (16%)	0/10 (0%)
**10–17**	1/54 (1.9%)	3/33 (9.1%)	1/29 (3.4%)	0/6 (0%)
**18–59**	2/161 (1.2%)	2/62 (3.2%)	3/60 (5.0%)	0/41 (0%)
**≥60**	4/63 (6.3%)	1/25 (4.0%)	0/18 (0%)	0/12 (0%)

Cells indicate the number of HUS cases out of the number of *E. coli* O157:H7 cases for a given age group and genotype. "Other" genotypes included *stx1a*-only, *stx1a2a2c*, *stx1a2c*, and *stx2c*-only. Abbreviations: CI, confidence interval; RD, risk difference; RR, relative risk

Table S2. Risk of HUS among *E. coli* O157 cases by genotype.

Genotype	Cases	HUS	Cumulative Incidence (95% CI)
stx1a	12	1	0.083 (0.002, 0.385)
stx1a2a	439	28	0.064 (0.043, 0.091)
stx1a2a2c	6	0	0 (0, 0.459)
stx1a2c	39	0	0 (0, 0.09)
stx2a	225	24	0.107 (0.070, 0.155)
stx2a2c	190	16	0.084 (0.049, 0.133)
stx2c	25	0	0 (0, 0.137)
**Total**	938	69	0.074(0.058, 0.092)

Abbreviations: CI, confidence interval; HUS, hemolytic uremic syndrome.

Table S3. Excess risk of HUS due to *stx2a* vs. other genotypes, among children <10 years old.

***stx2a* vs.**	**RD (95% CI)**		
	**Crude**	**Symptom-Adjusted**	**Fully Adjusted**
stx1a2a	0.041 (−0.048, 0.130)	0.036 (−0.051, 0.123)	0.034 (−0.054, 0.121)
stx2a2c	0.027 (−0.078, 0.131)	0.052 (−0.053, 0.158)	0.053 (−0.052, 0.159)
**Other**	0.131 (0.026, 0.237)	0.057 (−0.053, 0.167)	0.056 (−0.053, 0.165)

Symptom adjusted models are adjusted for blood in stool, vomiting, and fever. Fully adjusted models are adjusted for age, blood in stool, vomiting, and fever. Abbreviations: CI, confidence interval; RD, risk difference; RR, relative risk

Table S4. Excess risk of HUS due to *stx2a* without other *stx* genes among hospitalized patients.

***stx2a* (*N* = 94) vs.**	**N**	**RD (95% CI)**	**RR (95% CI)**
stx1a2a	177	0.072(−0.022, 0.165)	1.34(0.87, 2.05)
stx2a2c	85	0.041(−0.064, 0.147)	1.17(0.74, 1.86)
**Other**	20	0.101(−0.023, 0.224)	4.03(0.82, 19.70)

Models are adjusted for age, fever, and antibiotic treatment. Abbreviations: CI, confidence interval; RD, risk difference; RR, relative risk.

Table S5. Excess risk of HUS due to *stx2a* without other *stx* genes after correcting for potential gene loss.

***stx2a* (*N* = 185) vs.**	**N**	**RD (95% CI)**	**RR (95% CI)**
stx1a2a	448	0.052 (0, 0.104)	1.7 (1.04, 2.79)
stx2a2c	223	0.042 (−0.016, 0.1)	1.58 (0.92, 2.69)
**Other**	80	0.059 (0.009, 0.109)	4.44 (0.65, 30.12)

Models are adjusted for age, blood in stool, vomiting, and fever. Abbreviations: CI, confidence interval; RD, risk difference; RR, relative risk.

References

1. Tarr, G.A.M.; Shringi, S.; Phipps, A.I.; Besser, T.E.; Mayer, J.; Oltean, H.N.; Wakefield, J.; Tarr, P.I.; Rabinowitz, P. Geogenomic Segregation and Temporal Trends of Human Pathogenic *Escherichia coli* O157:H7, Washington, USA, 2005–2014(1). *Emerg. Infect. Dis.* **2018**, *24*, 32–39, doi:10.3201/eid2401.170851.
2. Jelacic, J.K.; Damrow, T.; Chen, G.S.; Jelacic, S.; Bielaszewska, M.; Ciol, M.; Carvalho, H.M.; Melton-Celsa, A.R.; O'Brien, A.D.; Tarr, P.I. Shiga toxin-producing *Escherichia coli* in Montana: Bacterial genotypes and clinical profiles. *J. Infect. Dis.* **2003**, *188*, 719–729, doi:10.1086/376999.
3. Klein, E.J.; Boster, D.R.; Stapp, J.R.; Wells, J.G.; Qin, X.; Clausen, C.R.; Swerdlow, D.L.; Braden, C.R.; Tarr, P.I. Diarrhea etiology in a Children's Hospital Emergency Department: A prospective cohort study. *Clin. Infect. Dis.* **2006**, *43*, 807–813, doi:10.1086/507335.

4. Tarr, G.A.M.; Shringi, S.; Oltean, H.N.; Mayer, J.; Rabinowitz, P.; Wakefield, J.; Tarr, P.I.; Besser, T.E.; Phipps, A.I. Importance of case age in the purported association between phylogenetics and hemolytic uremic syndrome in *Escherichia coli* O157:H7 infections. *Epidemiol. Infect.* **2018**, *146*, 1550–1555, doi:10.1017/S0950268818001632.

© 2019 by the authors. Submitted for possible open access publication under the terms and conditions of the Creative Commons Attribution (CC BY) license (http://creativecommons.org/licenses/by/4.0/).
